# Evaluation of [18F]-FDG-Based Hybrid Imaging Combinations for Assessment of Bone Marrow Involvement in Lymphoma at Initial Staging

**DOI:** 10.1371/journal.pone.0164118

**Published:** 2016-10-10

**Authors:** Ulrika Asenbaum, Richard Nolz, Georgios Karanikas, Julia Furtner, Ramona Woitek, Anton Staudenherz, Daniela Senn, Markus Raderer, Michael Weber, Ingrid Simonitsch-Klupp, Marius E. Mayerhoefer

**Affiliations:** 1 Department of Biomedical Imaging and Image-guided Therapy, Medical University of Vienna, Vienna, Austria; 2 Department of Internal Medicine I, Division of Oncology, Medical University of Vienna, Vienna, Austria; 3 Clinical Institute of Pathology, Medical University of Vienna, Vienna, Austria; Banner Alzheimer's Institute, UNITED STATES

## Abstract

The purpose of our study was to determine the value of different hybrid imaging combinations for the detection of focal and diffuse bone marrow infiltration in lymphoma. Patients with histologically proven lymphoma, who underwent both [18F]-FDG-PET/CT and whole-body MRI (including T1- and diffusion-weighted [DWI] sequences) within seven days, and a subsequent bone marrow biopsy, were retrospectively included. Three hybrid imaging combinations were evaluated: (1) [18F]-FDG-PET/CT; (2) [18F]-FDG-PET/T1; and (3) [18F]-FDG-PET/DWI. The presence of focal or diffuse bone marrow infiltration was assessed by two rater teams. Sensitivity, specificity, and accuracy for the detection of overall, focal, and diffuse bone marrow involvement were compared between the three hybrid imaging combinations. Overall, lymphomatous bone marrow involvement was found in 16/60 patients (focal, 8; diffuse, 8). Overall sensitivity, specificity, and accuracy were 81.3%, 95.5%, and 91.7% for [18F]-FDG-PET/CT; 81.3%, 97.7%, and 93.3% for [18F]-FDG-PET/T1; and 81.3%, 95.5%, and 91.7% for [18F]-FDG-PET/DWI. No statistically significant differences between the three imaging combinations were observed, based on overall bone marrow involvement, focal involvement, or diffuse involvement. The sensitivity of all three imaging combinations for detecting diffuse bone marrow involvement was only moderate (62.5% for all three combinations). Although the combination of [18F]-FDG-PET and T1-weighted MRI generally showed the best diagnostic performance for the detection of bone marrow involvement in lymphoma, it was not significantly superior to the two other hybrid imaging combinations. Since the sensitivity of all imaging combinations for the detection of diffuse bone marrow involvement was only moderate, bone marrow biopsy cannot be replaced by imaging as yet.

## Introduction

Multifocal or diffuse bone marrow involvement in lymphoma patients is a criterion for Ann Arbor stage IV disease, and often has a considerable effect on therapy and prognosis [1]. Bone marrow involvement is found in about 5–15% of patients with Hodgkin lymphoma (HL), and in 20–40% of patients with Non-Hodgkin lymphoma (NHL), depending on the histological subtype [2, 3]. Currently, 2-F-18-fluoro-2-deoxy-D-glucose-positron emission tomography/computed tomography ([18F]-FDG-PET/CT) is the recommended imaging method for initial staging and follow-up for the majority of lymphomas. Nevertheless, detection of bone marrow involvement—particularly diffuse marrow infiltration—on [18F]-FDG-PET/CT depends heavily on the histologic lymphoma subtype. Specifically, indolent lymphomas, such as follicular lymphomas are challenging in that regard. Thus, unilateral (or, more rarely, bilateral) blind bone marrow trephine biopsy (BMB) of the iliac crest is still a standard procedure in all lymphomas except HL [1]. However, BMB is associated with the small but recognized risks of hemorrhage or fracture, and is frequently regarded as painful, when performed without conscious sedation. Another limitation of this biopsy technique is the lack of image guidance, which can lead to false-negative results in cases of focal bone involvement. However, there are currently no guidelines, or sufficient data, that support the notion that use of image-guided biopsy, rather than standard iliac crest biopsy, has a clinically relevant effect on staging, or improves patient survival.

Whole-body magnetic resonance imaging (MRI) has shown promise as an alternative to [18F]-FDG-PET/CT for lymphoma staging, and possibly also for follow-up, particularly when diffusion-weighted imaging (DWI) sequences are included in the protocol [4–7]. DWI is a functional MRI technique that relies on the restriction of water movement in hypercellular tumors due to extracellular space narrowing, and thus, enables an indirect assessment of cell density. While DWI has been reported to increase the accuracy of MRI for the assessment of bone metastases from different tumors [8, 9], its value for the detection of bone marrow infiltration in lymphoma is still controversial [10, 11].

The aim of the present study was, therefore, to determine the value of different combinations of [18F]-FDG-PET with morphological or functional CT- or MRI-based imaging techniques, which may be combined in the form of hybrid imaging (i.e., PET/CT or PET/MRI), for the detection of bone marrow involvement in lymphoma. In this context, the question of whether [18F]-FDG-PET/MRI might be superior to [18F]-FDG-PET/CT for the assessment of diffuse bone marrow involvement received special attention.

## Materials and Methods

### Patients and Design

This retrospective single-center study of patients was approved by the local Institutional Review Board (Medical University Vienna) according to the Declaration of Helsinki. Informed consent was waived.A database search was performed to identify all patients with (1) histologically proven lymphoma and subtype classification according to the current classification of the World Health Organization (WHO) for hematologic and lymphoid malignancies, and who had (2) undergone both pre-therapeutic [18F]-FDG-PET/CT and additional whole-body MRI with DWI at 3.0 Tesla (in the course of previous, prospective studies, or for routine purposes), and in whom (3) a pre-therapeutic bone marrow biopsy and histological work-up had been performed in-house, by a reference pathologist specializing in hematological malignancies. The maximum allowed time interval between the first of the two imaging examinations (PET/CT or MRI) and bone marrow biopsy was one month. Exclusion criteria were a record of therapeutic interventions between [18F]-FDG-PET/CT and whole-body MRI, the presence of severe MRI artifacts, or an incomplete MR examination.

### Imaging

Whole-body [18F]-FDG-PET/CT was performed for routine diagnostic purposes using a 64-row multi-detector PET/CT scanner (Biograph TruePoint64; Siemens, Erlangen, GER). Glucose levels were measured before the examination, with a cut-off limit of 8 mmol/L (150 mg/dL). Imaging was performed 50–60 min following the intravenous administration of 300 MBq of [18F]-FDG. First, CT was performed after an intravenous injection of 100 mL of a tri-iodinated, non-ionic contrast medium (Iomeron 300; Bracco, Milan, ITA) at a rate of 2 mL/s, followed by a 50-mL saline flush. The following acquisition parameters were used: a tube voltage of 230 kV; a tube current of 120 mA; a collimation of 64 × 0.6 mm; a slice thickness of 3 mm with 2-mm increments; and a matrix of 512×512. CT was acquired in a breath-hold during (non-forced) expiration. Afterward, without changing the patient’s position, a whole-body PET acquisition was performed over five to six bed positions with 3 min/bed position. PET images were reconstructed using the iterative TrueX algorithm (Siemens, Erlangen, Germany), which incorporates a specific correction for the point-spread function in addition to commonly used correction factors. Four iterations per 21 subsets were used, with a matrix size of 168×168 and a transaxial field of view of 605 mm (pixel size 3.6 mm), and a section thickness of 5 mm. The attenuation correction was based on the CT maps. CT and PET data were co-registered, and image fusion was performed to generate color-coded images.

Whole-body MRI was performed on a 3Tesla MR scanner (TrioTim; Siemens, Erlangen, Germany). The study protocol included an established, commercially available, single-shot echo planar imaging (EPI)-based spectral adiabatic inversion recovery DWI sequence and a T1-weighted turbo spin-echo, or (in case of breathing difficulties) a fast gradient-echo (FLASH, fast low angle short) sequence. The DWI sequence was performed with b-values of 50 and 1000, a repetition time (TR)/ echo time (TE) of 5100/73ms, five averages, 86 phase encoding steps, a 192x115 matrix, and a slice thickness of 5mm with no gap. Axial images of the head and neck, chest, abdomen, and pelvis were sequentially obtained using a whole-body, phased-array surface coil for signal reception. Images were acquired during free breathing with a scan time of 4 minutes and 5 seconds per body region, except for the lower neck and chest, where respiratory gating was used with a prolonged scan time of 5 minutes and 2 seconds. Depending on the patients’ body height, four to five image stacks were necessary, with a total scan time between 20 and 25 min. Apparent diffusion coefficient (ADC) maps were generated automatically by the operational software supplied by the scanner manufacturer of the above-mentioned MR scanner. Axial T1-weighted sequences were obtained for better anatomical/morphological correlation, and to generate fused, color-coded DWI-MRI images. The T1-weighted, turbo spin-echo sequence was performed with a scan time of 1 minute 5 seconds; or alternatively, the fast T1-weighted gradient-echo sequence, with a scan time of 43 seconds.

### Bone marrow biopsy

Bone marrow biopsy (BMB) of the left iliac crest was performed by an experienced board-certified hematologist, without any form of imaging guidance, and before any form of systemic therapy was applied. A board-certified reference pathologist, who was blinded to all imaging data and results, analyzed all tissue samples.

### Image analysis

The following three imaging combinations were evaluated for the presence of bone marrow involvement in lymphoma: (1) [18F]-FDG-PET/CT; (2) [18F]-FDG-PET + T1-weighted MRI ([18F]-FDG-PET/T1); and (3) [18F]-FDG-PET + DWI ([18F]-FDG-PET/DWI).

For [18F]-FDG-PET/CT, focal bone marrow involvement was defined as the presence of one or more well-circumscribed areas of abnormal FDG uptake that was higher than the uptake in the liver, regardless of the appearance on CT. Similarly, diffuse bone marrow involvement in PET/CT was defined as visually elevated, diffuse FDG uptake (i.e., with ill-defined borders or confluent, patchy abnormal uptake), which was higher than the uptake of the liver, regardless of the appearance on CT.

For [18F]-FDG-PET/T1, the presence of one or more well-circumscribed focal areas, or a diffuse area of abnormal FDG uptake that was higher than the uptake in the liver, was rated as positive, provided that there were also corresponding ill-defined hypointense signal changes on T1 (lower signal than that of normal vertebral discs).

For [18F]-FDG-PET/DWI, the presence of one or more well-circumscribed focal areas, or a diffuse area of abnormal FDG uptake that was higher than the uptake in the liver, was rated as positive, provided that there was a corresponding focal or diffuse area of diffusion restriction (high signal intensity on the b-1000 image and corresponding low signal intensity on the co-registered ADC map). A focal area of diffusion restriction was also rated as positive in the absence of an increased FDG uptake.

All imaging combinations were reviewed on a Syngo workstation, using the dedicated “TrueD” software module (Siemens, Erlangen, Germany). Image interpretation was performed by two independent teams, which consisted of a board-certified radiologist (R.N., M.M.) and a board-certified nuclear medicine physician (G.K., A.S.) who specialized in oncological imaging. Readers were aware of the patient diagnosis of lymphoma, but were blinded to the histologic subtype. Imaging combinations were assessed in three reading sessions (first, [18F]-FDG-PET/CT; second, [18F]-FDG-PET/T1; third, [18F]-FDG-PET/DWI), with a time interval of two weeks between the reading sessions. Patient order was randomly assigned in each reading session for each reading team.

### Standard of reference

The consensus rating by an expert team, consisting of a senior radiologist, a senior nuclear medicine physician, and a senior pathologist, who had access to all imaging studies (including, if available, follow-up imaging and the results of BMB), was used as standard of reference. Focal bone marrow involvement was regarded as present when either the BMB result was positive, or when a focal FDG uptake, higher than that in the normal liver parenchyma, was present on the pre-therapeutic [18F]-FDG-PET/CT, and subsequently disappeared or was clearly lower (by at least 25%), on the follow-up [18F]-FDG-PET/CT. Diffuse bone marrow involvement was considered positive only in case of a positive BMB result, since diffuse FDG-uptake in PET may also be attributable to benign causes, such as inflammatory changes or elevated metabolism of hematopoietic tissue [12, 13].

### Statistical considerations

Statistical planning and analysis were performed by a statistician (M.W.), using the SPSS 22.0 software package for Microsoft Windows, (SPSS Inc., Chicago, lL, USA). Sensitivity, specificity, negative and positive predictive values, and accuracy, with their corresponding 95% confidence intervals for the detection of bone marrow involvement were calculated separately for [18F]-FDG-PET/CT, [18F]-FDG-PET/T1, and [18F]-FDG-PET/DWI, and independently for each of the two rater teams. Data analysis was also separately performed for overall, focal, and diffuse bone marrow involvement. Sensitivity, specificity, negative and positive predictive value, and accuracy were also calculated for BMB. McNemar tests were used for pairwise comparison of accuracies of the three imaging combinations, and kappa (κ) coefficients were used to assess interobserver agreement. The specified level of significance was 5% for all tests.

## Results

### Patients

Overall, 72 patients matched the inclusion criteria. Of these, six patients were excluded because of an incomplete MR examination, or artifacts; three were excluded because PET/CT had not been performed in-house; and three patients were excluded because the time interval between [18F]-FDG-PET/CT, MRI and BMB exceeded one month. Thus, 60 patients (32 females and 28 males; mean age, 51.2±16.7 years; age range, 20–80 years) were available for further analysis. Eighteen patients were diagnosed with Hodgkin lymphoma (HL), 16 with diffuse large B-cell lymphoma (DLBCL), 11 with follicular lymphoma (FL), six with extranodal marginal zone B-cell lymphoma of the mucosa-associated lymphoid tissue (MALT), four with mantle cell lymphoma (MCL), four with nodal marginal zone lymphoma (nMZL), and one with anaplastic large cell lymphoma (ALCL) (Table 1).

**Table 1:** Distribution of bone marrow involvement between the different lymphoma subtypes.

N = 60	Negative	Focal	Diffuse
**HL**	17	1	0
**DLBCL**	13	3	0
**FL**	4	3	4
**nMZL**	3	0	1
**MALT**	5	1	0
**MCL**	1	0	3
**ALCL**	1	0	0

HL, Hodgkin lymphoma; DLBCL, diffuse large B cell lymphoma; FL, follicular lymphoma; nMZL, nodal marginal zone lymphoma; MALT, extranodal marginal zone B-cell lymphoma of the mucosa-associated lymphoid tissue; MCL, mantle cell lymphoma; ALCL, anaplastic large cell lymphoma

### Standard of reference and BMB

**Fig 1:**
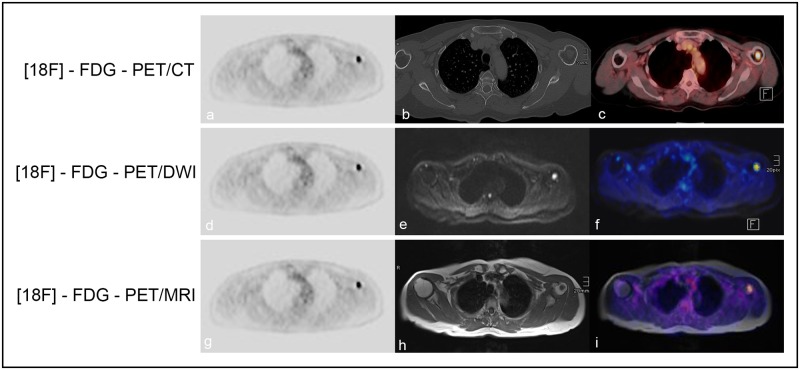
Focal bone marrow involvement. A 70-year-old female patient with histologically verified DLBCL and a focal bone marrow lesion in the left humerus. There is a perfect match between [18F]-FDG-PET and the fused color-coded PET/CT images (a-c); DWI and the fused color-coded [18F]-FDG-PET/DWI images (d-f); and [18F]-T1-weighted images and fused color-coded PET/T1 images (g-i), with regard to the detection of the focal lesion in the humerus.

**Fig 2:**
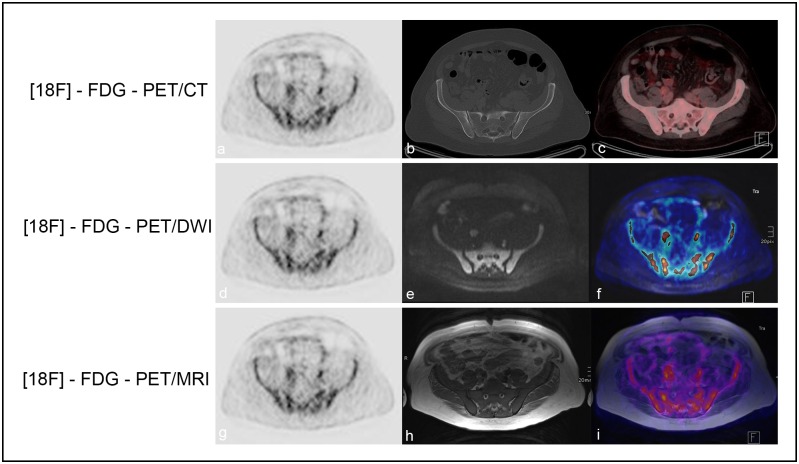
Diffuse bone marrow involvement. A 46-year-old male patient with histologically verified MCL, and diffuse bone marrow involvement proven by BMB and histology. There is a perfect match between [18F]-FDG-PET/CT and the fused color-coded PET/CT images (a-c); DWI and the fused color-coded [18F]-FDG-PET/DWI images (d-f); and [18F]-T1-weighted images and fused color-coded PET/T1 images (g-i), with regard to the detection of diffuse bone marrow involvement in the pelvic bones.

By consensus, bone marrow infiltration was considered present in 16/60 patients; 15/16 were diagnosed with an NHL, and one with an HL. Eight patients (seven NHL [three DLBCL, three FL, one MALT], one HL) showed focal bone marrow involvement, whereas the remaining eight patients with NHL (four FL, three MCL, one nMZL) showed diffuse bone marrow infiltration. Bone marrow biopsy was positive in 12/60 patients with a sensitivity, specificity, PPV, NPV, and accuracy of 75%, 100%, 100%, 91.7%, and 86.7%, respectively. Examples are given in Figs 1 and 2.

In patients with focal involvement, BMB was false-negative in 4/8 cases, with a sensitivity, specificity, PPV, NPV, and accuracy of 50%, 100%, 100%, 91.7%, and 92.3%, respectively. Due to the fact the BMB was mandatory for the diagnosis of diffuse bone marrow infiltration, BMB was positive in 8/8 patients.

### Performance of [18F]-FDG-PET/CT

Rater team 1 correctly evaluated bone marrow status on [18F]-FDG-PET/CT in 51/60 patients (85%), of whom 11 patients (six patients with focal, and five with diffuse affection) showed bone marrow involvement. Of the four false-positive patients, three had diffusely increased bone marrow uptake, but had a negative BMB. Five patients were false-negative (two patients with focal, and three with diffuse marrow affection). Overall, [18F]-FDG-PET/CT had a sensitivity, specificity, and accuracy of 68.8%, 90.9%, and 85.0%, respectively.

Rater team 2 correctly evaluated bone marrow status on [18F]-FDG-PET/CT in 55/60 patients (91.7%), whereas 13 patients were positive for bone marrow involvement (eight patients with focal, and five with diffuse affection). Two false-positive-rated patients showed diffusely increased bone marrow uptake, but were negative on BMB. The remaining three patients who were false-negative on [18F]-FDG-PET/CT showed a positive BMB result. Sensitivity, specificity, and accuracy for PET/CT were 81.3%, 95.5%, and 91.7%, respectively (Tables 2–4).

**Table 2:** Overall performance of the different imaging combinations for the detection of bone marrow involvement in lymphoma.

N = 60	Sensitivity	Specificity	PPV	NPV	Accuracy	Interrater κ
**Rater 1**						
**[18F]-FDG-PET/CT**	0.69 (0.41–0.88)	0.91 (0.77–0.97)	0.73 (0.45–0.91)	0.89 (0.75–0.96)	0.85 (0.74–0.92)	0.67 (*P*<0.001)
**[18F]-FDG-PET/T1**	0.69 (0.42–0.88)	0.93 (0.8–0.98)	0.79 (0.49–0.94)	0.89 (0.76–0.96)	0.87 (0.76–0.93)	0.83 (*P*<0.001)
**[18F]-FDG-PET/DWI**	0.81 (0.54–0.95)	0.93 (0.8–0.98)	0.81 (0.54–0.95)	0.93 (0.80–0.98)	0.90 (0.80–0.95)	0.88 (*P*<0.001)
**Rater 2**						
**[18F]-FDG-PET/CT**	0.81 (0.54–0.95)	0.96 (0.83–0.99)	0.87 (0.58–0.98)	0.93 (0.81–0.98)	0.92 (0.82–0.96)	-
**[18F]-FDG-PET/T1**	0.81 (0.54–0.95)	0.98 (0.87–1)	0.93 (0.64–1)	0.94 (0.81–0.98)	0.93 (0.84–0.97)	-
**[18F]-FDG-PET/DWI**	0.81 (0.54–0.95)	0.96 (0.83–0.99)	0.87 (0.58–0.98)	0.93 (0.81–0.98)	0.92 (0.82–0.96)	-
**BMB**	0.75 (0.47–0.92)	1.0 (0.9–1)	1.0 (0.7–1)	0.92 (0.79–0.97)	0.87 (0.76–0.93)	-

Values in parentheses represent 95% confidence intervals

PPV, positive predictive value; NPV, negative predictive value

**Table 3:** Focal bone marrow involvement—diagnostic performance of the different imaging combinations.

N = 52	Sensitivity	Specificity	PPV	NPV	Accuracy	Interrater κ
**Rater 1**						
**[18F]-FDG-PET/CT**	0.75 (0.36–0.96)	0.91 (0.77–0.97)	0.60 (0.27–0.86)	0.95 (0.83–0.99)	0.89 (0.77–0.95)	0.76 (*P*<0.001)
**[18F]-FDG-PET/T1**	0.88 (0.47–0.99)	0.93 (0.8–0.98)	0.70 (0.35–0.92)	0.98 (0.86–1.0)	0.92 (0.82–0.97)	0.81 (*P*<0.001)
**[18F]-FDG-PET/DWI**	1.0 (0.60–1.0)	0.93 (0.8–0.98)	0.73 (0.39–0.93)	1.0 (0.89–1.0)	0.94 (0.84–0.98)	0.94 (*P*<0.001)
**Rater 2**						
**[18F]-FDG-PET/CT**	1.0 (0.6–1)	0.96 (0.83–0.99)	0.80 (0.44–0.97)	1.0 (0.9–1.0)	0.96 (0.87–0.99)	-
**[18F]-FDG-PET/T1**	1.0 (0.60–1.0)	0.98 (0.87–1.0)	0.89 (0.51–0.99)	1.0 (0.90–1.0)	0.98 (0.90–1.0)	-
**[18F]-FDG-PET/DWI**	1.0 (0.6–1.0)	0.96 (0.83–0.99)	0.80 (0.44–0.97)	1.0 (0.90–1.0)	0.96 (0.87–0.99)	-
**BMB**	0.5 (0.17–0.83)	1.0 (0.90–1.0)	1.0 (0.40–1.0)	0.92 (0.79–0.97)	0.92 (0.82–0.97)	-

Values in parentheses represent 95% confidence intervals

PPV, positive predictive value; NPV, negative predictive value

**Table 4:** Diffuse bone marrow involvement—diagnostic performance of the different imaging combinations.

N = 52	Sensitivity	Specificity	PPV	NPV	Accuracy	Interrater κ
**Rater 1**						
**[18F]-FDG-PET/CT**	0.63 (0.26–0.90)	0.91 (0.77–0.97)	0.56 (0.23–0.85)	0.93 (0.80–0.98)	0.87 (0.75–0.93)	0.56 (*P*<0.001)
**[18F]-FDG-PET/T1**	0.50 (0.17–0.83)	0.93 (0.80–0.98)	0.57 (0.20–0.88)	0.91 (0.78–0.97)	0.87 (0.75–0.93)	0.74 (*P*<0.001)
**[18F]-FDG-PET/DWI**	0.63 (0.26–0.90)	0.93 (0.80–0.98)	0.63 (0.26–0.90)	0.93 (0.80–0.98)	0.89 (0.77–0.95)	0.77 (*P*<0.001)
**Rater 2**						
**[18F]-FDG-PET/CT**	0.63 (0.26–0.90)	0.96 (0.83–0.99)	0.71 (0.30–0.95)	0.93 (0.81–0.98)	0.90 (0.79–0.96)	-
**[18F]-FDG-PET/T1**	0.63 (0.26–0.90)	0.98 (0.87–1.0)	0.83 (0.37–0.99)	0.94 (0.81–98)	0.92 (0.82–0.97)	-
**[18F]-FDG-PET/DWI**	0.63 (0.26–0.90)	0.96 (0.83–0.99)	0.71 (0.30–0.95)	0.93 (0.81–0.98)	0.90 (0.79–0.96)	-
**BMB**	1.0 (0.60–1.0)	1.0 (0.90–1.0)	1.0 (0.60–1.0)	1.0 (0.90–1.0)	1.0 (0.93–1.0)	-

Values in parentheses represent 95% confidence intervals

PPV, positive predictive value; NPV, negative predictive value

### Performance of [18F]-FDG-PET/T1

Rater team 1 correctly evaluated bone marrow status on [18F]-FDG-PET/T1 in 54/60 patients (86.7%). Three patients were false-positive, all of whom had signs of diffuse marrow infiltration. All of the three false-negative patients had a positive BMB. Sensitivity, specificity, and accuracy for [18F]-FDG-PET/T1 were 68.8%, 93.2%, and 86.7%, respectively.

Rater team 2 also assessed bone marrow status correctly in 56/60 patients (93.3%). Three patients were false-negative, and the single false-positive patient again showed signs suggestive of diffuse marrow infiltration at imaging. Thus, sensitivity, specificity, PPV, NPV, and accuracy for [18F]-FDG-PET/T1 were 100%, 97.7%, and 98.1%, respectively (Tables 2–4).

### Performance of [18F]-FDG-PET/DWI

Rater team 1 correctly evaluated bone marrow status on PET/DWI in 54/60 patients (90%). Three patients were false-positive, all of whom had with signs of diffuse marrow infiltration. All of the three false-negative patients had a positive BMB. Sensitivity, specificity, and accuracy for [18F]-FDG-PET/DWI were 81.3%, 93.2%, and 90.0%, respectively.

Rater team 2 also assessed bone marrow status correctly in 55/60 patients (91.7%). Three patients were false-negative, and the two false-positive patients again showed signs suggestive of diffuse marrow infiltration at imaging. Thus, sensitivity, specificity, and accuracy for [18F]-FDG-PET/DWI were 81.3%, 95.5%, and 91.7%, respectively (Tables 2–4).

### Comparison of accuracies and interrater agreement

McNemar tests did not reveal statistically significant differences between any of the three imaging combinations in terms of accuracy (*P*>0.05). The kappa values between the two rater teams ranged from 0.67 to 1.0 (overall), and from 0.76 to 1.0 for focal involvement. However, for diffuse infiltration, there was only a moderate interrater agreement, with kappa values between 0.56 and 0.74 (Tables 2–4).

## Discussion

Our study is, to our knowledge, the first that specifically compares three hybrid imaging combinations—[18F]-FDG-PET/CT, [18F]-FDG-PET/T1, and [18F]-FDG-PET/DWI—for the detection of bone marrow involvement in lymphoma patients. This topic is of relevance due to the fact that PET/MRI is an emerging technique whose diagnostic value is yet to be established, and due to the role of bone marrow involvement in the Ann Arbor staging system, i.e., assignment of patients to stage IV [14]. Our study results indicate that [18F]-FDG-PET/MR—regardless of whether [18F]-FDG-PET is combined with T1-weighted MRI, or with DWI—provides a level of diagnostic performance similar to that of the current hybrid imaging standard technique, [18F]-FDG-PET/CT.

Nevertheless, despite a lack of statistically significant difference, between the three imaging combinations, the combinations of [18F]-FDG-PET with T1-weighted MRI, and DWI, were slightly superior to [18F]-FDG-PET/CT, in terms of accuracy (Tables 2–4). This finding is of interest due to the availability of PET/MR scanners that offer combinations of both PET and MRI in a single examination. However, our results do not justify, or even suggest, the replacement of [18F]-FDG-PET/CT by [18F]-FDG-PET/MRI based on its performance in bone marrow assessment, particularly in view of the considerably higher costs associated with PET/MR. In addition, the most frequently used method of PET attenuation correction for PET/MRI typically relies on two-point VIBE Dixon T1-weighted sequences, where cortical bone is assigned to the soft tissues, which, in turn, leads to a systematic underestimation of standardized uptake values [15]. Only in young patients, for which exposure to ionizing radiation should be as low as possible in order to minimize the risk of secondary radiation-induced cancer, can [18F]-FDG-PET/MRI be considered a better choice than PET/CT, since our results, while unable to demonstrate a superiority, also did not demonstrate an inferiority, with regard to bone marrow status assessment, compared to [18F]-FDG-PET/CT.

As expected, due to the presence of patients with focal bone marrow involvement in our study population, unilateral iliac crest bone marrow biopsy (which is the current diagnostic standard for bone marrow evaluation in lymphoma patients), showed a clearly lower sensitivity than each of the three imaging combinations. However, for the assessment of diffuse marrow infiltration, none of the three imaging combinations was able to provide a diagnostic performance that was comparable to that of bone marrow biopsy. These findings provide further proof that imaging may have a complementary role in such patients, and could thus be useful chiefly for guiding biopsies. For the [18F]-FDG-PET-based combinations, one major factor that may lead to false-positive findings is the fact that a diffuse FDG tracer uptake is frequently seen in hematopoietic tissue, or is attributable to reactive / inflammatory marrow changes [12, 13]; here, the histologic subtype should probably be taken into account, because it is well-known that diffuse bone marrow infiltration is mainly found in NHL, particularly in the indolent subtypes. This was also evident when looking at our own data, where a diffuse marrow infiltration was observed exclusively in indolent NHL. The latter may also be responsible for our false-negative [18F]-FDG-PET findings, because indolent NHL frequently show a rather low glycolytic activity, compared to the aggressive subtypes [16]. Here, dual or delayed time-point [18F]-FDG-PET, which has shown improved visualization of MALT lymphomas in a previous study [17], may possibly be helpful.

The question of whether DWI is a vital part of whole-body MRI protocols in the context of lymphomatous bone marrow involvement has previously been investigated by Kwee et al., who found no difference in sensitivity between whole-body MRI with, and whole-body MRI without, DWI [10]. The sensitivities of MRI with/without DWI were generally unsatisfactory in their study, for which [18F]-FDG-PET/CT was not available, but which used BMB as the only standard of reference. In view of the latter, the authors pointed out that the actual sensitivity of MRI with/without DWI may be higher, due to focal lesions missed by standard iliac crest biopsy. Ribrag et al. compared the diagnostic performance of BMB, whole-body MRI, and [18F]-FDG-PET/CT for bone marrow infiltration in 43 patients with aggressive lymphomas, and observed good agreement between MRI and [18F]-FDG-PET/CT, with patient-based sensitivities of 100% and 100%, respectively, and lesion-based sensitivities of 83% and 96%, respectively; both imaging techniques also identified more patients with bone marrow involvement than BMB [18]. However, these authors used a STIR and a T1-weighted MR sequence, but no DWI, and they did not differentiate between focal and diffuse involvement by imaging. Adams et al. also compared whole-body MRI to [18F]-FDG-PET for the detection of bone marrow involvement in 116 patients with aggressive and indolent lymphomas, using BMB as the reference standard [19]. Again, in their study, there was no significant difference in terms of marrow involvement detection between the two imaging modalities; while they used DWI as part of their MRI protocol, they did not evaluate its role and potential benefits. In our own study, which combined [18F]-FDG-PET with DWI, and with T1-weighted MRI, no clear benefit arose from the use of DWI, which suggests that, for bone marrow assessment on PET/MRI, DWI may be omitted.

Our study had several limitations. First, due to our strict inclusion criteria (e.g., regarding the maximum time interval between imaging and bone marrow biopsy), the number of patients with histological proof of bone marrow involvement was limited. Due to the retrospective character of our study, patients with focal bone marrow involvement that was missed by biopsy could not be assigned to guided biopsy to obtain definitive proof of lymphomatous involvement; however, it is highly unlikely that lesions that met our non-histology-based criteria for bone marrow involvement—i.e., a therapy-induced reduction of FDG uptake—were actually benign. Second, [18F]-FDG-PET and the two MR sequences (T1-weighted and DWI) were not obtained on an integrated hybrid PET/MRI scanner, but on two separate devices, which may affect conclusions regarding the comparative performances of PET/CT and PET/MRI, due to the above-mentioned differences in terms of attenuation correction for PET. Nevertheless, the CT-based attenuation correction used in our study is the current standard technique for PET, and is considered to be more reliable than MRI-based attenuation correction. Finally, subgroup analyses between the different NHL lymphoma types, with regard to sensitivity, specificity, and accuracy were not possible, due to the small number of patients in each group.

In conclusion, the results of our study indicate that, compared to [18F]-FDG-PET/CT, the use of [18F]-FDG-PET/MRI may provide only a minor degree of improvement, with regard to the assessment of bone marrow status in patients with lymphoma, but not a statistically significant advantage. Notably, in the context of [18F]-FDG-PET/MRI, DWI does not provide an advantage, compared to T1-weighted MRI, for the assessment of bone marrow involvement. Since the sensitivity of [18F]-FDG-PET/CT and the two PET/MRI combinations was only moderate for the detection of diffuse bone marrow involvement, bone marrow biopsy cannot be replaced by imaging as yet.

## Supporting Information

S1 Data Statistical Data. (PDF)

## References

[1] Cheson BD, Fisher RI, Barrington SF, Cavalli F, Schwartz LH, Zucca E, et al Recommendations for initial evaluation, staging, and response assessment of Hodgkin and non-Hodgkin lymphoma: the Lugano classification. Journal of clinical oncology: official journal of the American Society of Clinical Oncology. 2014;32(27):3059–68. 10.1200/JCO.2013.54.8800 . 25113753 PMC4979083

[2] Coller BS, Chabner BA, Gralnick HR. Frequencies and patterns of bone marrow involvement in non-Hodgkin lymphomas: observations on the value of bilateral biopsies. Am J Hematol. 1977;3:105–19. Epub 1977/01/01. 10.1002/ajh.2830030201 . 602932

[3] Conlan MG, Bast M, Armitage JO, Weisenburger DD. Bone marrow involvement by non-Hodgkin's lymphoma: the clinical significance of morphologic discordance between the lymph node and bone marrow. Nebraska Lymphoma Study Group. Journal of clinical oncology: official journal of the American Society of Clinical Oncology. 1990;8(7):1163–72. Epub 1990/07/01. . 1694234 10.1200/JCO.1990.8.7.1163

[4] Mayerhoefer ME, Karanikas G, Kletter K, Prosch H, Kiesewetter B, Skrabs C, et al Evaluation of Diffusion-Weighted MRI for Pretherapeutic Assessment and Staging of Lymphoma: Results of a Prospective Study in 140 Patients. Clin Cancer Res. 2014;20(11):2984–93. 10.1158/1078-0432.Ccr-13-3355. WOS:000337157000017. 24696320

[5] Mayerhoefer ME, Karanikas G, Kletter K, Prosch H, Kiesewetter B, Skrabs C, et al Evaluation of Diffusion-Weighted Magnetic Resonance Imaging for Follow-up and Treatment Response Assessment of Lymphoma: Results of an 18F-FDG-PET/CT-Controlled Prospective Study in 64 Patients. Clin Cancer Res. 2015;21(11):2506–13. 10.1158/1078-0432.Ccr-14-2454. WOS:000357335800014. 25733598

[6] Lin C, Luciani A, Itti E, El-Gnaoui T, Vignaud A, Beaussart P, et al Whole-body diffusion-weighted magnetic resonance imaging with apparent diffusion coefficient mapping for staging patients with diffuse large B-cell lymphoma. European radiology. 2010;20(8):2027–38. Epub 2010/03/24. 10.1007/s00330-010-1758-y . 20309558

[7] Abdulqadhr G, Molin D, Astrom G, Suurkula M, Johansson L, Hagberg H, et al Whole-body diffusion-weighted imaging compared with FDG-PET/CT in staging of lymphoma patients. Acta radiologica. 2011;52(2):173–80. 10.1258/ar.2010.100246 . 21498346

[8] Takenaka D, Ohno Y, Matsumoto K, Aoyama N, Onishi Y, Koyama H, et al Detection of bone metastases in non-small cell lung cancer patients: comparison of whole-body diffusion-weighted imaging (DWI), whole-body MR imaging without and with DWI, whole-body FDG-PET/CT, and bone scintigraphy. Journal of magnetic resonance imaging: JMRI. 2009;30(2):298–308. Epub 2009/07/25. 10.1002/jmri.21858 . 19629984

[9] Grankvist J, Fisker R, Iyer V, Frund ET, Simonsen C, Christensen T, et al MRI and PET/CT of patients with bone metastases from breast carcinoma. European journal of radiology. 2012;81(1):e13–8. 10.1016/j.ejrad.2010.10.024 . 21227614

[10] Kwee TC, Fijnheer R, Ludwig I, Quarles van Ufford HM, Uiterwaal CS, Bierings MB, et al Whole-body magnetic resonance imaging, including diffusion-weighted imaging, for diagnosing bone marrow involvement in malignant lymphoma. Br J Haematol. 2010;149(4):628–30. Epub 2010/02/05. 10.1111/j.1365-2141.2010.08093.x . 20128795

[11] Jiang XX, Yan ZX, Song YY, Zhao WL. A pooled analysis of MRI in the detection of bone marrow infiltration in patients with malignant lymphoma. Clinical radiology. 2013;68(3):e143–53. 10.1016/j.crad.2012.11.002 . 23245271

[12] Salaun PY, Gastinne T, Bodet-Milin C, Campion L, Cambefort P, Moreau A, et al Analysis of 18F-FDG PET diffuse bone marrow uptake and splenic uptake in staging of Hodgkin's lymphoma: a reflection of disease infiltration or just inflammation? European journal of nuclear medicine and molecular imaging. 2009;36(11):1813–21. 10.1007/s00259-009-1183-0 . 19499219

[13] Murata Y, Kubota K, Yukihiro M, Ito K, Watanabe H, Shibuya H. Correlations between 18F-FDG uptake by bone marrow and hematological parameters: measurements by PET/CT. Nuclear medicine and biology. 2006;33(8):999–1004. 10.1016/j.nucmedbio.2006.09.005 . 17127173

[14] Lister TA, Crowther D, Sutcliffe SB, Glatstein E, Canellos GP, Young RC, et al Report of a committee convened to discuss the evaluation and staging of patients with Hodgkin's disease: Cotswolds meeting. Journal of clinical oncology: official journal of the American Society of Clinical Oncology. 1989;7(11):1630–6. . 2809679 10.1200/JCO.1989.7.11.1630

[15] Eiber M, Takei T, Souvatzoglou M, Mayerhoefer ME, Furst S, Gaertner FC, et al Performance of whole-body integrated 18F-FDG PET/MR in comparison to PET/CT for evaluation of malignant bone lesions. Journal of nuclear medicine: official publication, Society of Nuclear Medicine. 2014;55(2):191–7. 10.2967/jnumed.113.123646 . 24309383

[16] Paes FM, Kalkanis DG, Sideras PA, Serafini AN. FDG PET/CT of extranodal involvement in non-Hodgkin lymphoma and Hodgkin disease. Radiographics. 2010;30(1):269–91. 10.1148/rg.301095088 . 20083598

[17] Mayerhoefer ME, Giraudo C, Senn D, Hartenbach M, Weber M, Rausch I, et al Does Delayed-Time-Point Imaging Improve 18F-FDG-PET in Patients With MALT Lymphoma?: Observations in a Series of 13 Patients. Clinical nuclear medicine. 2016;41(2):101–5. 10.1097/RLU.0000000000001005 26402137 PMC4703065

[18] Ribrag V, Vanel D, Leboulleux S, Lumbroso J, Couanet D, Bonniaud G, et al Prospective study of bone marrow infiltration in aggressive lymphoma by three independent methods: whole-body MRI, PET/CT and bone marrow biopsy. European journal of radiology. 2008;66(2):325–31. Epub 2007/07/27. 10.1016/j.ejrad.2007.06.014 . 17651934

[19] Adams HJ, Kwee TC, Vermoolen MA, de Keizer B, de Klerk JM, Adam JA, et al Whole-body MRI for the detection of bone marrow involvement in lymphoma: prospective study in 116 patients and comparison with FDG-PET. European radiology. 2013;23(8):2271–8. Epub 2013/04/18. 10.1007/s00330-013-2835-9 . 23591618

