# Supplementary material for: Evaluation of [18F]-FDG-Based Hybrid Imaging Combinations for Assessment of Bone Marrow Involvement in Lymphoma at Initial Staging
Source: PLoS One. 2016 Oct 10;11(10):e0164118. doi: 10.1371/journal.pone.0164118 (PMC5056699; doi:10.1371/journal.pone.0164118)
Supplement: S1 Data — (PDF) [file pone.0164118.s001.pdf]

PatID	**Consensus**	**Histo**	**PETCTr1**	**PET+DWIr1**	**PETT1r1**	**PETCTr2**
1	1	0	1	1	1	1
2	1	0	0	1	1	1
3	0	0	0	0	0	0
4	0	0	0	0	0	0
5	0	0	0	0	0	0
6	0	0	0	0	0	0
7	0	0	0	0	0	0
8	1	1	0	1	1	1
9	0	0	0	0	0	0
10	0	0	0	0	0	0
11	1	1	1	1	1	1
12	0	0	0	0	1	0
13	0	0	0	0	0	0
14	0	0	0	0	0	0
15	0	0	1	0	0	0
16	0	0	0	0	0	0
17	0	0	0	0	0	0
18	1	1	1	1	1	1
19	0	0	0	0	0	0
20	0	0	0	0	0	0
21	0	0	0	0	0	0
22	1	1	1	1	1	1
23	0	0	1	1	0	1
24	1	0	1	1	1	1
25	0	0	0	0	0	0
26	0	0	0	0	0	0
27	0	0	0	0	0	0
28	1	1	1	1	1	1
29	0	0	1	1	0	0
30	1	1	1	1	0	0
31	1	1	0	0	0	0
32	0	0	0	0	0	0
33	0	0	0	0	0	0
34	0	0	0	0	1	0
35	0	0	0	0	0	0
36	0	0	0	0	0	0
37	0	0	0	0	0	0
38	0	0	0	0	0	0
39	0	0	0	0	0	0
40	0	0	0	0	0	0
41	0	0	0	0	0	0
42	0	0	0	0	0	0
43	1	1	1	0	0	0
44	0	0	0	0	0	0
45	0	0	0	0	0	0

46	0	0	0	0	0	0
47	0	0	1	1	1	1
48	1	0	1	1	1	1
49	1	1	1	1	1	1
50	0	0	0	0	0	0
51	0	0	0	0	0	0
52	1	1	1	1	1	1
53	0	0	0	0	0	0
54	1	1	0	1	0	1
55	0	0	0	0	0	0
56	1	1	0	0	0	1
57	0	0	0	0	0	0
58	0	0	0	0	0	0
59	0	0	0	0	0	0
60	0	0	0	0	0	0

PET+DWIr2	PETT1r2	Consensusfd	Histo	PETCTr1fd	PET+DWIr1fd	T1+Petr1fd
1	1	1	0	1	1	1
1	1	1	0	0	1	1
0	0	0	0	0	0	0
0	0	0	0	0	0	0
0	0	0	0	0	0	0
0	0	0	0	0	0	0
0	0	0	0	0	0	0
1	1	2	1	0	2	2
0	0	0	0	0	0	0
0	0	0	0	0	0	0
1	1	2	1	2	2	2
0	0	0	0	0	0	2
0	0	0	0	0	0	0
0	0	0	0	0	0	0
0	0	0	0	1	0	0
0	0	0	0	0	0	0
0	0	0	0	0	0	0
1	1	1	1	1	1	1
0	0	0	0	0	0	0
0	0	0	0	0	0	0
0	0	0	0	0	0	0
1	1	2	1	2	2	2
1	0	0	0	2	2	0
1	1	1	0	1	1	1
0	0	0	0	0	0	0
0	0	0	0	0	0	0
0	0	0	0	0	0	0
1	1	1	1	1	1	1
0	0	0	0	2	2	0
0	0	2	1	2	2	0
0	0	2	1	0	0	0
0	0	0	0	0	0	0
0	0	0	0	0	0	0
0	1	0	0	0	0	2
0	0	0	0	0	0	0
0	0	0	0	0	0	0
0	0	0	0	0	0	0
0	0	0	0	0	0	0
0	0	0	0	0	0	0
0	0	0	0	0	0	0
0	0	0	0	0	0	0
0	0	0	0	0	0	0
0	0	2	1	2	0	0
0	0	0	0	0	0	0
0	0	0	0	0	0	0

0	0	0	0	0	0	0
1	0	0	0	2	2	2
1	1	1	0	1	1	1
1	1	2	1	2	2	2
0	0	0	0	0	0	0
0	0	0	0	0	0	0
1	1	1	1	1	1	1
0	0	0	0	0	0	0
1	1	1	1	0	1	0
0	0	0	0	0	0	0
1	1	2	1	0	0	0
0	0	0	0	0	0	0
0	0	0	0	0	0	0
0	0	0	0	0	0	0
0	0	0	0	0	0	0

PETCTr2fd	PET+DWIr2fd	T1+PETr2fd
1	1	1
1	1	1
0	0	0
0	0	0
0	0	0
0	0	0
0	0	0
2	2	2
0	0	0
0	0	0
2	2	2
0	0	0
0	0	0
0	0	0
0	0	0
0	0	0
0	0	0
1	1	1
0	0	0
0	0	0
0	0	0
2	2	2
2	2	0
1	1	1
0	0	0
0	0	0
0	0	0
1	1	1
0	0	0
0	0	0
0	0	0
0	0	0
0	0	0
0	0	2
0	0	0
0	0	0
0	0	0
0	0	0
0	0	0
0	0	0
0	0	0
0	0	0
0	0	0
0	0	0
0	0	0

0	0	0
2	2	0
1	1	1
2	2	2
0	0	0
0	0	0
1	1	1
0	0	0
1	1	1
0	0	0
2	2	2
0	0	0
0	0	0
0	0	0
0	0	0
